# Distribution of Carnivore protoparvovirus 1 in free-living leopard cats (*Prionailurus bengalensis chinensis*) and its association with domestic carnivores in Taiwan

**DOI:** 10.1371/journal.pone.0221990

**Published:** 2019-09-03

**Authors:** Chen-Chih Chen, Ai-Mei Chang, Takayuki Wada, Mei-Ting Chen, Yun-Shan Tu

**Affiliations:** 1 Institute of Wildlife Conservation, College of Veterinary Medicine, National Pingtung University of Science and Technology, Pingtung, Taiwan; 2 Research Center for Animal Biologics, National Pingtung University of Science and Technology, Pingtung, Taiwan; 3 Graduate Institute of Animal Vaccine Technology, College of Veterinary Medicine, National Pingtung University of Science and Technology, Pingtung, Taiwan; 4 Department of International Health, Institute of Tropical Medicine, Nagasaki University, Nagasaki, Japan; 5 Leopard Cat Association of Taiwan, Miaoli, Taiwan; University of Lincoln, UNITED KINGDOM

## Abstract

Carnivore protoparvovirus 1 (CPPV-1) is widespread among free-living carnivores, and CPPV-1 infection may directly or indirectly impact on the population of endangered carnivore species. In this study, we used molecular screening of viral capsid protein 2 (VP2) from 2015 to 2017, to assess the prevalence of CPPV-1 infection in 9 live-trapped (LT) and 17 vehicle collision (VC)-affected free-living leopard cats (*Prionailurus bengalensis chinensis*). In addition, we conducted the phylogenetic analysis to evaluate the possible transmission of CPPV-1 between domestic carnivores and leopard cats. We identified the circulation of feline parvovirus and variants of canine parvovirus (CPV), including CPV-2a, CPV-2b, and CPV-2c, in the free-living leopard cat population. The partial sequences of different variants of *VP2* obtained from the leopard cats were identical with those obtained from the domestic dogs and cats in Taiwan. Our result suggested that CPPV-1 was currently transmitted between domestic carnivores and leopard cats in Taiwan. A plan of conservation measures based on vaccination program for domestic carnivores, strict controls on populations of free-living dogs and cats and limiting road development only to low-risk areas for leopard cats should be encouraged.

## Introduction

The leopard cat (*Prionailurus bengalensis chinensis*) is an endangered felid species, which is distributed in East, Southeast, and South Asia [1]. It was previously commonly distributed in the lowland habitats throughout the island of Taiwan [2, 3]. However, owing to the island-wide decline in the population of this species, it was listed as an endangered species under the Wildlife Conservation Act in Taiwan in 2009 [4]. Currently, the distribution of Taiwanese leopard cats is restricted to small areas in 3 counties, namely Miaoli, Nantou, and Taichung City, in Central Taiwan. Studies in the Miaoli County suggested that road kill, habitat fragmentation and degradation, illegal trapping, and poisoning are the major threats to the sustainability of the leopard cat population [5].

Road kill is considered a factor threatening the sustainability of wild mammal populations, particularly that of populations of endangered species in rural areas [6, 7]. Based on our records, at least 50 road kill cases of leopard cats were found from 2012 to 2017 in Taiwan. In addition to the direct impact of vehicle collision on endangered species, development of road systems could facilitate the domestic carnivores, such as dog and cat, to invade into the fragmented habitat and transmit the exotic pathogens to the native species[8]. Free-roaming dogs and cats are commonly noticed around the habitat of leopard cats in Miaoli. Stray and free-roaming domestic animals, particularly dogs and cats, can adversely affect wildlife conservation through predation, competition, hybridization, and disease transmission [9, 10]. Furthermore, studies indicated that the movements of free-roaming dogs in area are primarily following the road systems [11, 12]. These interactions may increase the transmission of pathogens between dogs and leopard cats, such as carnivore protoparvovirus 1 (CPPV-1), a worldwide distributed virus which commonly be found to infect both domestic and wild carnivores [13–15].

CPPV-1 belonging to the genus *Protoparvovirus* of the family Parvoviridae, can cause life-threatening pathogenic infections in many carnivores [13]. According to the International Committee on Taxonomy of Viruses, numerous phylogenetically similar viral strains that infect carnivore species are classified under CPPV-1, including feline parvovirus, variants of canine parvovirus (CPV including CPV-2a, CPV-2b, and CPV-2c), mink enteritis virus, and raccoon parvovirus [16]. Infections of variants of CPV-2a, CPV-2b, CPV-2c, and feline parvovirus among domestic and wild felids have been commonly documented [13, 17, 18]. In Taiwan, infection with CPV-2a has been reported in apparently healthy captive leopard cats [19, 20]. Furthermore, CPV-2b and CPV-2c were isolated from leopard cats in Vietnam [20]. Felids infected with feline parvovirus exhibit acute depression, diarrhea, vomiting, and panleukopenia [21]. No evidence supports hypothesis that the disease are induced by CPV-2 derived variants (2a, 2b, and 2c) in leopard cats. However, in several studies, domestic cats infected with CPV-2a and CPV-2c developed a similar clinical signs of feline parvovirus [22–24]. Although disease induced by CPPV-1 infection was more commonly found in the juvenile or sub-adult individual of domestic dogs and cats, adult individuals with severe clinical signs of CPPV-1 infection were recorded [23, 25, 26]. There was an increasing number of documents reporting severe CPPV-1 enteritis in adult dogs [26, 27].

CPV-2 derived variants and feline parvovirus have been documented to infect domestic dogs and cats in Taiwan, respectively [28–30]. Therefore, transmission of CPPV-1 between domestic dogs, cats, and leopard cats is highly possible. Nevertheless, the effects of parvovirus infection on the leopard cat population may be overlooked.

In the present study, free-living leopard cats were screened for CPPV-1 infection by using a nested polymerase chain reaction (nested PCR) method. Furthermore, the nested PCR amplicons were sequenced for phylogenetic analysis for evaluating the possible transmission between sympatric carnivores and identifying the possible source of parvovirus. Our hypothesis was that the CPPV-1 has been transmitted between domestic carnivores and leopard cats. Therefore, the phylogeny of each variant of CPPV-1would shows high similarity between domestic carnivores and leopard cats.

## Materials and methods

### Study area

**Fig 1:**
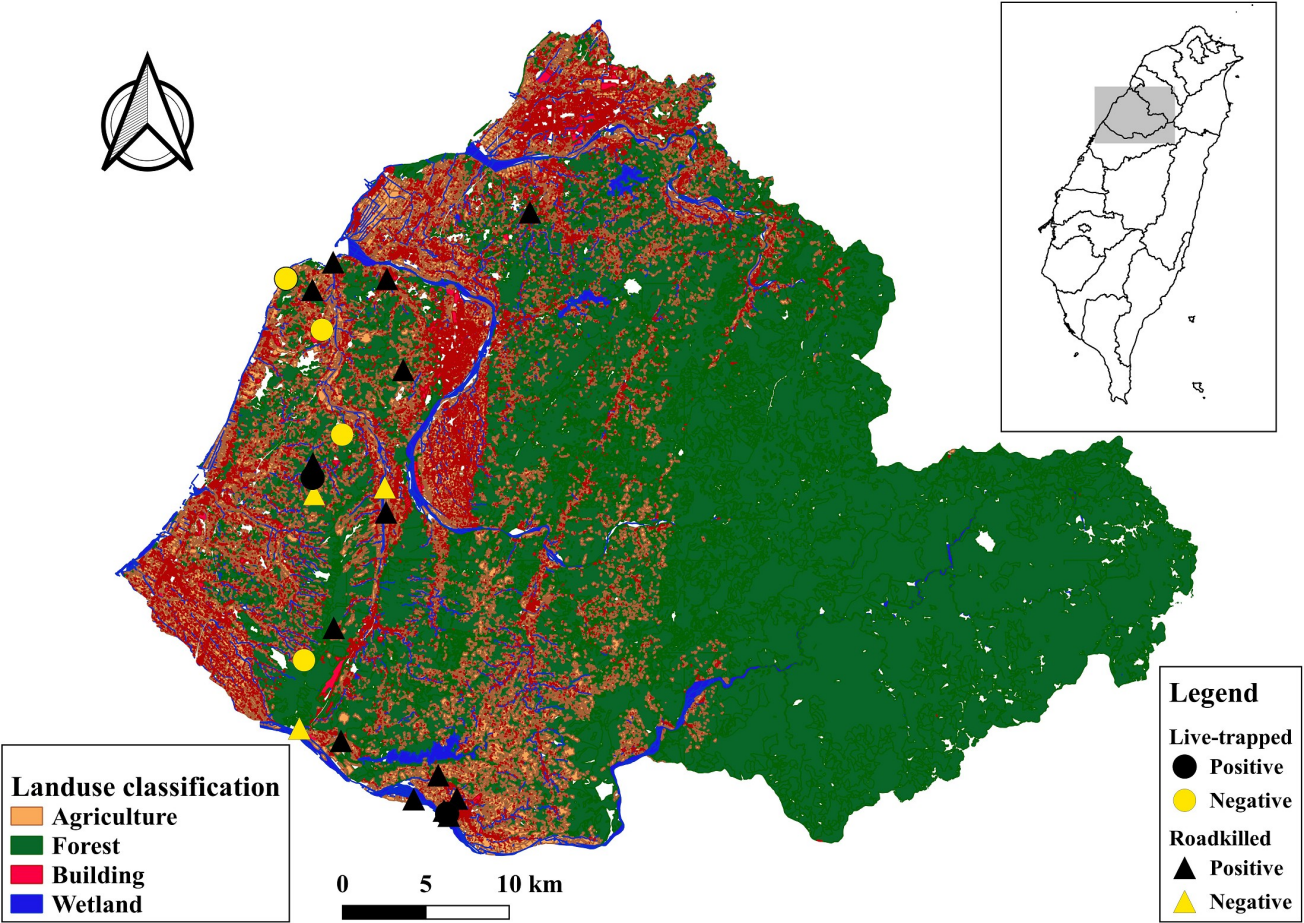
Sampling sites of leopard cats in Miaoli County. Circles and triangles denote live-trapped (LT) and vehicle-collision (VC)-affected leopard cats, respectively. Black and yellow denote Carnivore protoparvovirus 1 (CPPV-1) positive and CPPV-1 negative leopard cats, respectively.

All the leopard cats sampled were from Miaoli County in northwestern Taiwan (Fig 1). The sampling area has a hilly landscape with an elevation of less than 320 m above sea level. The total area of Miaoli County is 1820 km^2^, which consists of 1245.3 km^2^ of forests (68.8%), 291.2 km^2^ of agricultural land (16.1%), and 132.6 km^2^ of man-made construction (7.3%) (Fig 1). A well-developed road system, which includes a primary road (approximately 25 m wide), secondary roads (approximately 10 m wide), and tertiary roads (about 5 m wide), and human encroachment have fragmented the wildlife habitat in this rural area. The region has a subtropical climate with hot and wet summers, and cold and dry winters. The wet season lasts from March to September (mean monthly rainfall = 293 mm; mean monthly temperature = 25.4°C), while the dry season lasts from October to February (mean monthly rainfall = 72 mm; mean monthly temperature = 19.2°C) [31].

Although an estimate of the population of the stray or free-roaming dogs and cats was not available, they were commonly observed and were sympatric with the leopard cats in the study area [32].

### Sample collection

Samples were collected from leopard cats between 2015 and 2017. Free-living leopard cats were trapped for radio telemetry tracking or relocation of the leopard cats that invaded poultry farms, denoted as live trapped (LT) individuals. The trapping sites selected for free-living leopard cats were based on systematically auto-triggered camera survey for leopard cat distribution in each township area and farmer reported invasion of leopard cats into poultry farms. Permission for this study was issued by the Forest Bureau (Permit no.: COA, Forestry Bureau, 1061702029). We used steel-mesh box traps (108-Rigid Trap, Tomahawk Live Trap, LLC., Hazelhurst, Wisconsin, USA) baited with live quails. The trapped leopard cats were anesthetized by a veterinarian by using a mixture of medetomidine hydrochloride (50 μg/kg) and tiletamine HCl/zolazepam HCl (2 mg/kg). The procedures for leopard cat trapping, anesthesia administration, and sample collection were approved by Institutional Animal Care and Use Committee of National Pingtung University of Science and Technology (Approval no.: NPUST-106-014).

The collection of carcasses of vehicle collision (VC)-affected individuals were based on the public report of the site of VC, and collected and submitted by the county government of Miaoli for additional necropsy and sample collection.

We recorded sex and age for each leopard cat. Although there was no standard criterion of age classification for a leopard cat, we roughly categorized age of each individual into juvenile, sub-adult, adult and old adult based on the dentition. The criteria of age classification were deciduous dentition for juvenile, permanent dentition but not fully growth for sub-adult, fully growth of permanent dentition to mild abrasion of canine teeth for young adult, as well as moderate to severe abrasion of canine teeth for old adult.

We collected whole blood samples and rectal swabs of the LT leopard cats and spleen tissue and small intestine or rectal swabs of the VC-affected leopard cats for further nested PCR diagnosis. Whole blood and rectal swab samples of free-roaming dogs and cats were collected by a veterinarian of the Miaoli Animal Care and Health Office in the same study area. The samples of sympatric domestic carnivores were collected before their transfer to the rescue center to avoid the possible CPPV-1 infection in the rescue center. The samples from the dogs and cats were used to compare the DNA sequences between CPPV-1 isolated from domestic carnivores and leopard cats. We recorded the global positioning system (GPS) locations of all the LT leopard cats or those that were found dead, as well as the township locations of the dogs and cats for further spatial analysis.

From 2015 to 2017, we collected samples from 26 leopard cats of which 9 were LT and 17 were VC-affected individuals (S1 Table) in the western Miaoli County (Fig 1).

### PCR screening and phylogenetic analysis of the CPPV-1

We performed nested PCR for CPPV-1 screening using consensus primers that targeted *VP2*, which is the gene that codes for the outer capsid protein of the CPPV-1. The primers were designed by Steinel et al. [33]. In the first round of nested PCR, the forward primer M10 (5′-ACACATACATGGCAAACAAATAGA-3′) and reverse primer M11 (5′-ACTGGTGGTACATTATTTAATGCAG-3′) were used. In the second round, the forward primer M13 (5′-AAATAGAGCATTGGGCTTACCACCATTTTT-3′) and reverse primer M14 (5′-ATTCCTGTTTTACCTCCAATTGGATCTGTT-3′). Total DNA was extracted from the whole blood, spleen, and small intestine samples by using a DNeasy blood and tissue kit (Qiagen, Valencia, CA) and from the rectal swabs by using a QIAamp DNA Stool Mini Kit (Qiagen, Valencia, CA); the manufacturer’s instructions were followed for both types of samples. The conditions of nested PCR amplification mainly followed the protocol described by Steinel et al. [33], with minor modifications. Briefly, viral templates were amplified in a 20-μL reaction mixture, which contained PCR buffer (1.5 mM MgCl_2_, each deoxynucleoside triphosphate at a concentration of 200 M, and Hot-StarTaq Master Mix [Qiagen, Valencia, CA]), 0.2 μM of each PCR primer, and 2 μL of the DNA templates. The amplification procedure was as follows: 15 min at 95°C; 35 cycles of 30 s at 94°C, 30 s at 47°C, and 60 s at 72°C, and a final extension for 7 min at 72°C. The second round of nested PCR was performed using the same conditions. The expected size of the nested PCR product was 482 bp.

The PCR amplicons were sequenced in an ABI377 sequencer by using an ABI PRISM dye-terminator cycle sequencing ready reaction kit with Amplitaq DNA polymerase (Perkin-Elmer, Applied Biosystems). To search for sequences similar to those of the amplicons, a BLAST search was conducted using GenBank with the nt/nr database available on the BLAST website (BLAST; https://blast.ncbi.nlm.nih.gov/Blast.cgi). In addition, the viral strains isolated from leopard cats and domestic animals were classified according to the amino acid variation in *VP2* [17].

The nucleotide sequences were aligned with CLUSTALW [34] in the software MEGA version 6 [35]. The maximum-likelihood method [36] was used to model the phylogenetic relationship among the various CPPV-1 strains isolated from the leopard cats, dogs, and cats. Prior to the construction of a maximum-likelihood tree, the most suitable model was determined using MEGA 6.0. Consequently, the Tamura three-parameter model was selected on the basis of the lowest Bayesian information criterion value [37]. Finally, a phylogenetic tree was constructed using 1000 bootstrap iterations. To estimate evolutionary divergence, we grouped the CPPV-1 sequences according to source (leopard cats, domestic dogs, and cats) in the study area and compared them with sequences retrieved from the nucleotide database (NCBI GenBank). The database sequences had been isolated from domestic dogs and cats in other regions of Taiwan. All CPPV-1 sequences registered in GenBank after 2014, except the feline parvovirus sequences, were retrieved for this study. In total, 42 sequences (414 bp) were included for phylogenetic analysis (S3 Table). We constructed a phylogenetic tree by using the partial nucleotide sequences of *VP2* from the 17 leopard cat isolations, 14 domestic dog and cat isolations, and 22 sequences retrieved from GenBank.

## Results

### Phylogenetic analysis of the CPPV-1 strains circulating in the leopard cat, domestic dog, and domestic cat populations

**Fig 2:**
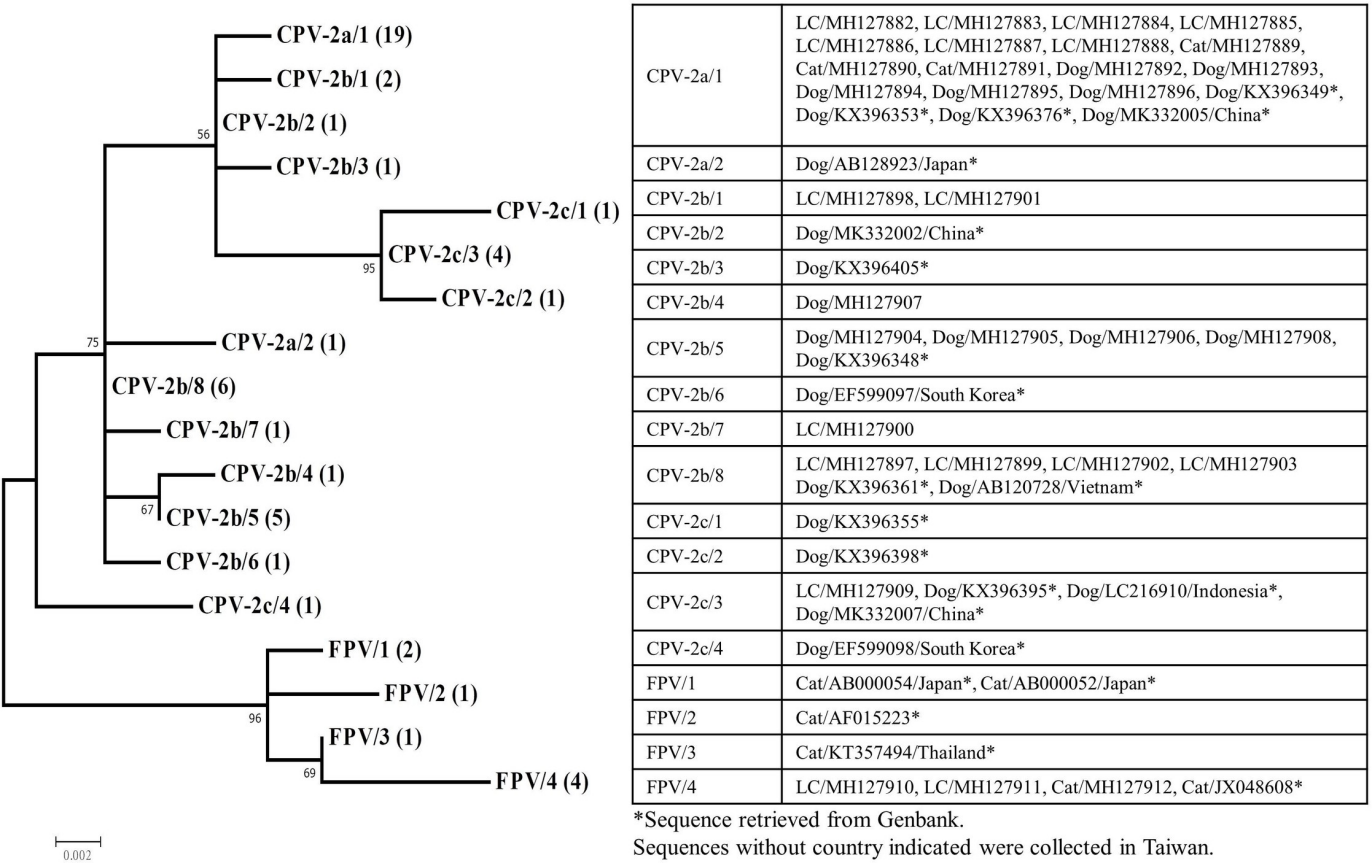
Molecular phylogenetic relationship of the partial *VP2* sequences of the Carnivore protoparvovirus 1 detected by PCR from leopard cats, domestic dogs, domestic cats, and sequences accessed from GenBank. The bootstrap value is shown next to the node with 1,000 replicates. Each variants and sequence type is labeled followed by the number of identical sequences within each group (e.g., CPV-2a/1 (19), indicating that the sequence type 1 of variants CPV-2a contains 19 identical sequences). Identical sequences in each group were listed in the table.

The screening of the samples collected from the 26 leopard cats revealed that the overall prevalence of CPPV-1 infection was 65.4% (95% CI 47.1%–83.7%). However, the prevalence of the 9 LT leopard cats was 33.3% and that in the 17 VC-affected leopard cats was 82.4% (Table 1). Based on the partial VP2 amino acid sequences obtained from the 17 CPPV-1 positive leopard cats, we determined that 7, 7, 1, and 2, leopard cats were infected with CPV-2a, CPV-2b, CPV-2c, and feline parvovirus, respectively (S2 Table). The partial VP2 amino acid and nucleotide sequences of CPV-2a were identical among some of the leopard cats, domestic dogs, and domestic cats. For CPV-2b, we found that Tyr at position 324 was substituted by an Ile in two samples collected from the leopard cats. In addition, a substitution of Asn to Lys at position of 321 was found in all the samples collected from domestic dogs infected with CPV-2b but not in those from the leopard cats. Only one sample of CPV-2c was obtained from the leopard cats in our study and was identical with one of the genotypes currently circulating in Taiwan (S3 Table). The sequences of CPV-2a, CPV-2c, and feline parvovirus included in the analysis were grouped into distinct clusters based on the variants (Fig 2). A unique cluster of CPV-2b from the leopard cat was found, which was different from the other CPV-2b sub-clusters observed in wild carnivores and domestic carnivores.

**Table 1:** Samples from live-trapped or vehicle-collision-affected leopard cats and the result of parvovirus screening and mean road coverage in the home range.

Type of animal analyzed^1^	No. Individuals	Parvovirus^2^	Prevalence	95% CI^3^	
				lower	upper
LT	9	3/9	33.3%	2.53%	64.13%
VC	17	14/17	82.4%	64.23%	100%

^1^LT: live-trapped leopard cats; VC: vehicle-collision-affected leopard cats.

^2^Results of parvovirus screening, positive/total individuals.

^3^CI: confidence interval.

## Discussion

In this study, circulation of CPPV-1, including the variants of feline parvovirus, CPV-2a, CPV-2b, and CPV-2c, was identified in the free-living leopard cat population. Except a distinct subclade of CPV-2b isolated from the leopard cats, the partial sequence of the variants of *VP2* isolated from the leopard cats were identical with those isolated from the domestic dogs and cats. This study is the first to report CPPV-1 infection in free-living leopard cats, but CPPV-1 infection has been previously reported in captive leopard cats [19] and various carnivores [15, 33, 38].

The nucleotide sequences of the CPPV-1 isolates suggested a high likelihood that variants of CPPV-1 had been transmitted between the domestic animals and the leopard cats. Cross-species transmission of CPPV-1 between domestic and free-living carnivores has been demonstrated or suspected in various countries [15, 38]. Allison et al. [39] found that the amino acid at VP2 position 300 is a key determinant of the host range. The receptor-binding ability and infectivity of VP2 position 300 mutants differ substantially. Therefore, mutations at VP2 position 300 affect the host range and resistance of a host to infection by the virus. The amino acid at VP2 position 300 of CPV-variants isolated in our study was glycine (S2 Table), which infects the Canidae and Felidae species. In addition, the amino acid at VP2 position 300 of the feline parvovirus strain isolated in our study was alanine, which infects the Felidae species [39]. We found a distinct genotype of CPV-2b isolated from the leopard cats. This result might imply the evolutionary isolation of the genotype in the leopard cat population. However, owing to the limitation of sample size, the analysis of the distribution of this distinct CPV-2b genotype was inconclusive. Furthermore, the amino acid at VP2 position 300 of this CPV-2b genotype was glycine, which indicated the possibility of transmission between the domestic dogs and leopard cats [39–41]. The primary reservoir of CPPV-1 was not possible to determine based on the results of our study. Nevertheless, the leopard cat is a critically endangered species in Taiwan and sustained CPPV-1 transmission in this low-density population is questionable [42]. We considered the stray dogs and cats, which exhibited the highest abundance among carnivores in study area, as the primary reservoirs.

The distribution of free-roaming dogs and cats in our study area was similar to that in other rural areas in Taiwan [43, 44]. In addition to a high population density, the vaccination and neutering coverage of free-roaming dogs and cats is usually low in Taiwan [45]. Groups of free-roaming dogs are active in areas with well-developed road systems [11, 12]; this aggravates the transmission of pathogens between dogs as well as the flow of pathogens into the habitat of leopard cats. The flow of pathogens is highly plausible, particularly for stable pathogens such as CPPV-1, which can retain their infectiousness in the environment for a long period [15]. In this study, we were not able to analyse the association between density of free-roaming domestic carnivores and CPPV-1 infection in leopard cats due to lacking distribution information on free-roaming domestic carnivores. Future studies should examine the influence of the density of free ranging domestic carnivores.

Our study revealed that free ranging leopard cats and domestic carnivores often have opportunity to interact and transmit CPPV-1 between each other. We strongly recommend establishment of efforts to manage of CPPV-1 in the leopard cat habitat with an emphasis on vaccination programs and population control measures for free-roaming dogs and cats. Additionally, previous studies have indicated that because of antigenic differences among CPPV-1 variants, new vaccines that also provide protection against the CPV-2c variant must be developed [26, 46].

## Supporting information

S1 Table Individual information for leopard cats collected in our study and the result of carnivore protoparvovirus screening. (DOCX)

S2 Table Multiple alignment of partial VP2 amino acid sequence of Carnivore protoparvovirus 1 isolated from leopard cats, domestic dogs and cats, and sequences downloaded from the National Center for Biotechnology Information. The species and virus strains are listed and the accession numbers are presented in parentheses.
(DOCX)

S3 Table DNA Sequences of partial *VP2* included in this study. Sequences were isolated from the leopard cats, domestic dogs and cats, and retrieved from Genbank with accession numbers provided in parenthesis.
(XLS)
