# Supplementary material for: Distribution of Carnivore protoparvovirus 1 in free-living leopard cats (Prionailurus bengalensis chinensis) and its association with domestic carnivores in Taiwan
Source: PLoS One. 2019 Sep 3;14(9):e0221990. doi: 10.1371/journal.pone.0221990 (PMC6719846; doi:10.1371/journal.pone.0221990)
Supplement: S1 Table — (DOCX) [file pone.0221990.s001.docx]

S1 Table. Individual information for leopard cats collected in our study and the result of carnivore protoparvovirus screening. LT/VC: live-trapped/vehicle collision.

| Individual Code | LT/VC | Sampling Date | Parvovirus screening | | | | Age | Sex |
| --- | --- | --- | --- | --- | --- | --- | --- | --- |
|  |  |  | Spleen | Intestine | Whole blood | Rectal swab |  |  |
| 2015011401 | LT | 2015/1/14 | - | - | Negative | Negative | Subadult | Male |
| 2015011402 | LT | 2015/1/14 | - | - | Negative | Negative | Subadult | Female |
| 2015011403 | LT | 2015/1/14 | - | - | Negative | Negative | Subadult | Male |
| 2015092001 | LT | 2015/9/20 | - | - | Negative | Negative | Young adult | Male |
| 2015110601 | LT | 2015/11/6 | - | - | Positive | Negative | Subadult | Female |
| 2016092901 | LT | 2016/9/29 | - | - | Negative | Negative | Young adult | Female |
| 2017110701 | LT | 2017/11/6 | - | - | Positive | Positive | Subadult | Male |
| 2017071201 | LT | 2017/7/12 | - | - | Negative | Negative | Subadult | Male |
| 2017112501 | LT | 2017/11/24 | - | - | Positive | Negative | Juvenile | Female |
| C2015011901 | VC | 2015/1/19 | Negative | Positive | - | - | Young adult | Male |
| C2015030901 | VC | 2015/3/9 | Negative | Negative | - | - | Young adult | Male |
| C2015061801 | VC | 2015/6/18 | Negative | Negative | - | - | Young adult | Female |
| C2015100901 | VC | 2015/10/9 | Positive | Negative | - | Negative | Young adult | Female |
| C2015110501 | VC | 2015/11/5 | Positive | Positive | - | - | Young adult | Female |
| C2015110502 | VC | 2015/11/5 | Negative | Positive | - | - | Young adult | Male |
| C2015110503 | VC | 2015/11/5 | Positive | Positive | - | - | Young adult | Female |
| C2015111901 | VC | 2015/11/19 | Positive | Positive | - | - | Young adult | Male |
| C2016010401 | VC | 2015/1/4 | Positive | Negative | - | - | Young adult | Male |
| C2016012801 | VC | 2016/1/28 | Positive | Negative | - | - | Young adult | Female |
| C2016030101 | VC | 2016/3/1 | Positive | Positive | - | - | Young adult | Female |
| C2016112701 | VC | 2016/11/27 | Positive | Negative | - | - | Young adult | Male |
| C2016120301 | VC | 2016/12/3 | None | Positive | - | - | Subadult | Male |
| C2017012901 | VC | 2017/1/27 | Negative | Negative | - | - | Young adult | Male |
| C2017090801 | VC | 2017/9/8 | Positive | Positive | - | Positive | Young adult | Male |
| C2017100201 | VC | 2017/10/2 | Positive | Positive | - | Positive | Juvenile | Male |
| C2017110701 | VC | 2017/11/7 | Positive | Negative | - | Positive | Young adult | Male |
