# Supplementary material for: Distribution of Carnivore protoparvovirus 1 in free-living leopard cats (Prionailurus bengalensis chinensis) and its association with domestic carnivores in Taiwan
Source: PLoS One. 2019 Sep 3;14(9):e0221990. doi: 10.1371/journal.pone.0221990 (PMC6719846; doi:10.1371/journal.pone.0221990)
Supplement: S2 Table — The species and virus strains are listed and the accession numbers are presented in parentheses. (DOCX) [file pone.0221990.s002.docx]

S2 Table. Multiple alignment of partial VP2 amino acid sequence of Carnivore protoparvovirus 1 isolated from leopard cats, domestic dogs and cats, and sequences downloaded from the National Center for Biotechnology Information. The species and virus strains are listed and the accession numbers are presented in parentheses.

| **Isolations** | **Amino acid position of VP2 gene** | | | | | | | | | | | | |
| --- | --- | --- | --- | --- | --- | --- | --- | --- | --- | --- | --- | --- | --- |
|  | **300** | **305** | **321** | **323** | **324** | **339** | **353** | **370** | **383** | **410** | **413** | **420** | **426** |
| P.bengalensis/c2015100901/CPV-2a | **G** | **Y** | **N** | **N** | **I** | **S** | **F** | **Q** | **Q** | **P** | **D** | **F** | **N** |
| P.bengalensis/2015110601/CPV-2a | . | . | . | . | . | . | . | . | . | . | . | . | . |
| P.bengalensis/c2016010401/CPV-2a | . | . | . | . | . | . | . | . | . | . | . | . | . |
| P.bengalensis/c2016030101/CPV-2a | . | . | . | . | . | . | . | . | . | . | . | . | . |
| P.bengalensis/2017100201/CPV-2a | . | . | . | . | . | . | . | . | . | . | . | . | . |
| P.bengalensis/2017112501/CPV-2a | . | . | . | . | . | . | . | . | . | . | . | . | . |
| P.bengalensis/2017110701/CPV-2a | . | . | . | . | . | . | . | . | . | . | . | . | . |
| 952/Cat/CPV-2a | . | . | . | . | . | . | . | . | . | . | . | . | . |
| 953/Cat/CPV-2a | . | . | . | . | . | . | . | . | . | . | . | . | . |
| 954/Cat/CPV-2a | . | . | . | . | . | . | . | . | . | . | . | . | . |
| 1019/Dog/CPV-2a | . | . | . | . | . | . | . | . | . | . | . | . | . |
| 1026/Dog/CPV-2a | . | . | . | . | . | . | . | . | . | . | . | . | . |
| 1036/Dog/CPV-2a | . | . | . | . | . | . | . | . | . | . | . | . | . |
| 1037/Dog/CPV-2a | . | . | . | . | . | . | . | . | . | . | . | . | . |
| 1038/Dog/CPV-2a | . | . | . | . | . | . | . | . | . | . | . | . | . |
| CPV-2a(KX396349) | . | . | . | . | . | . | . | . | . | . | . | . | . |
| CPV-2a(KX396353) | . | . | . | . | . | . | . | . | . | . | . | . | . |
| CPV-2a(KX396376) | . | . | . | . | . | . | . | . | . | . | . | . | . |
| P.bengalensis/c2015011901/CPV-2b | . | . | . | . | Y | . | . | . | . | . | . | . | D |
| P.bengalensis/c2015111901/CPV-2b | . | . | . | . | . | . | . | . | . | . | . | . | D |
| P.bengalensis/c2015110501/CPV 2b | . | . | . | . | Y | . | . | . | . | . | . | . | D |
| P.bengalensis/c2015110502/CPV-2b | . | . | . | . | Y | N | . | . | . | . | . | . | D |
| P.bengalensis/c2015110503/CPV-2b | . | . | . | . | . | . | . | . | . | . | . | . | D |
| P.bengalensis/c2016012801/CPV-2b | . | . | . | . | Y | . | . | . | . | . | . | . | D |
| P.bengalensis/c2016112701/CPV-2b | . | . | . | . | Y | . | . | . | . | . | . | . | D |
| 1020/Dog/CPV-2b | . | . | K | . | Y | . | . | . | . | . | . | . | D |
| 1023/Dog/CPV-2b | . | . | K | . | Y | . | . | . | . | . | . | . | D |
| 1025/Dog/CPV-2b | . | . | K | . | Y | . | . | . | . | . | . | . | D |
| 1027/Dog/CPV-2b | . | . | K | . | Y | . | . | . | . | . | N | . | D |
| 1028/Dog/CPV-2b | . | . | K | . | Y | . | . | . | . | . | . | . | D |
| CPV-2b(KX396348) | . | . | K | . | Y | . | . | . | . | . | . | . | D |
| CPV-2b(KX396361) | . | . | . | . | Y | . | . | . | . | . | . | . | D |
| P.bengalensis/2017090801/CPV-2c | . | . | . | . | . | . | . | R | . | . | . | . | E |
| CPV-2c(KX396355) | . | . | . | . | . | . | . | R | R | L | . | . | E |
| CPV-2c(KX396395) | . | . | . | . | . | . | . | R | . | . | . | . | E |
| CPV-2c(KX396398) | . | . | . | . | . | . | . | R | . | . | . | S | E |
| P.bengalensis/2016120301/FPV^1^ | A | D | . | D | Y | . | . | . | . | . | . | . | . |
| P.bengalensis/c2017110701/FPV | A | D | . | D | Y | . | . | . | . | . | . | . | . |
| 1018/Cat/FPV | A | D | . | D | Y | . | . | . | . | . | . | . | . |
| FPV(JX048608) | A | D | . | D | Y | . | . | . | . | . | . | . | . |
| FPV(AF015223) | A | D | . | D | Y | . | L | . | . | . | . | . | . |

FPV: feline parvovirus
